# Supplementary figures and images for: The effect of temperature on host patch exploitation by an egg parasitoid
Source: PLoS One. 2021 Jul 21;16(7):e0254750. doi: 10.1371/journal.pone.0254750 (PMC8294483; doi:10.1371/journal.pone.0254750)

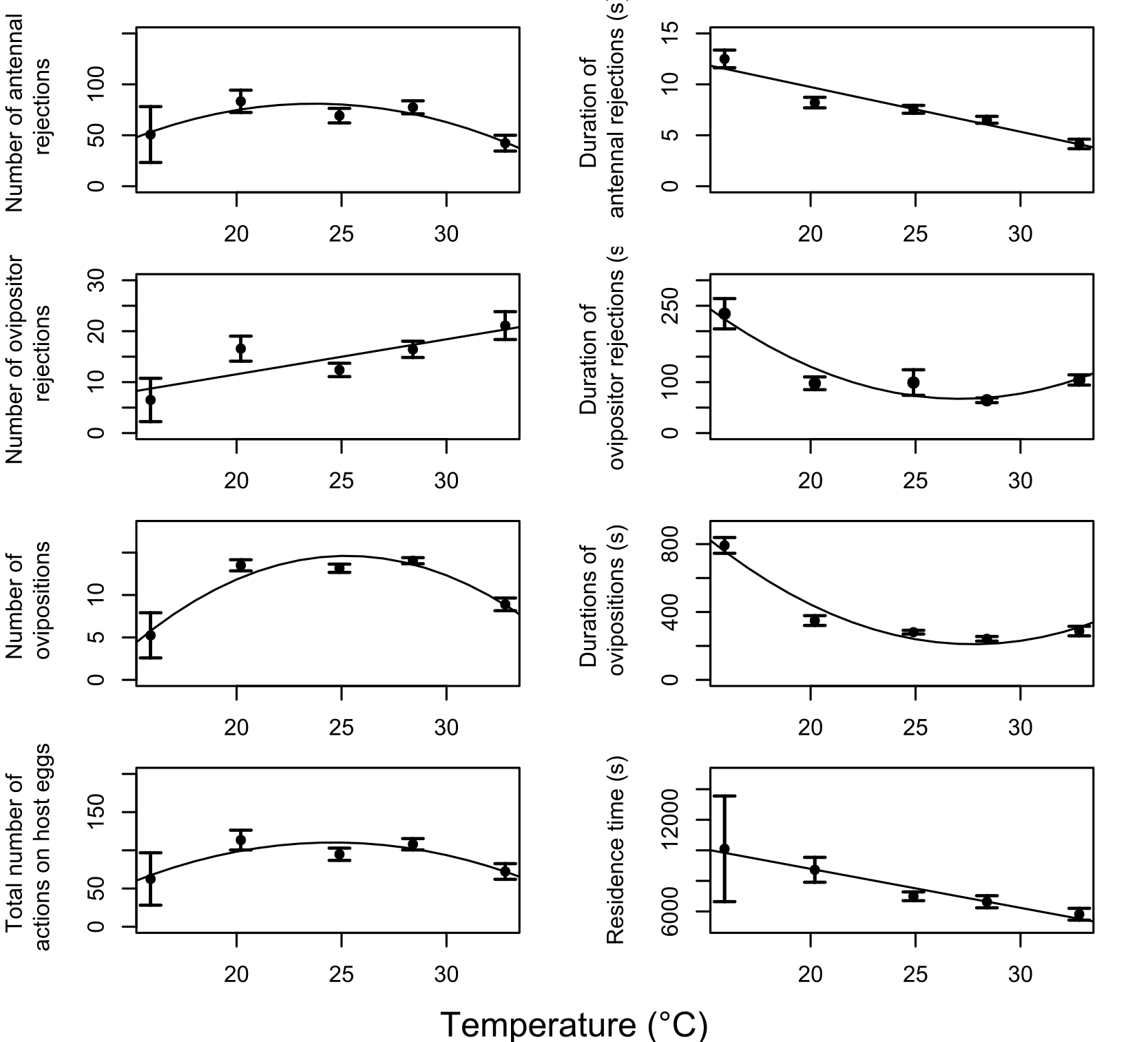

Supplement: S1 Fig — (TIF) [file pone.0254750.s001.tif]

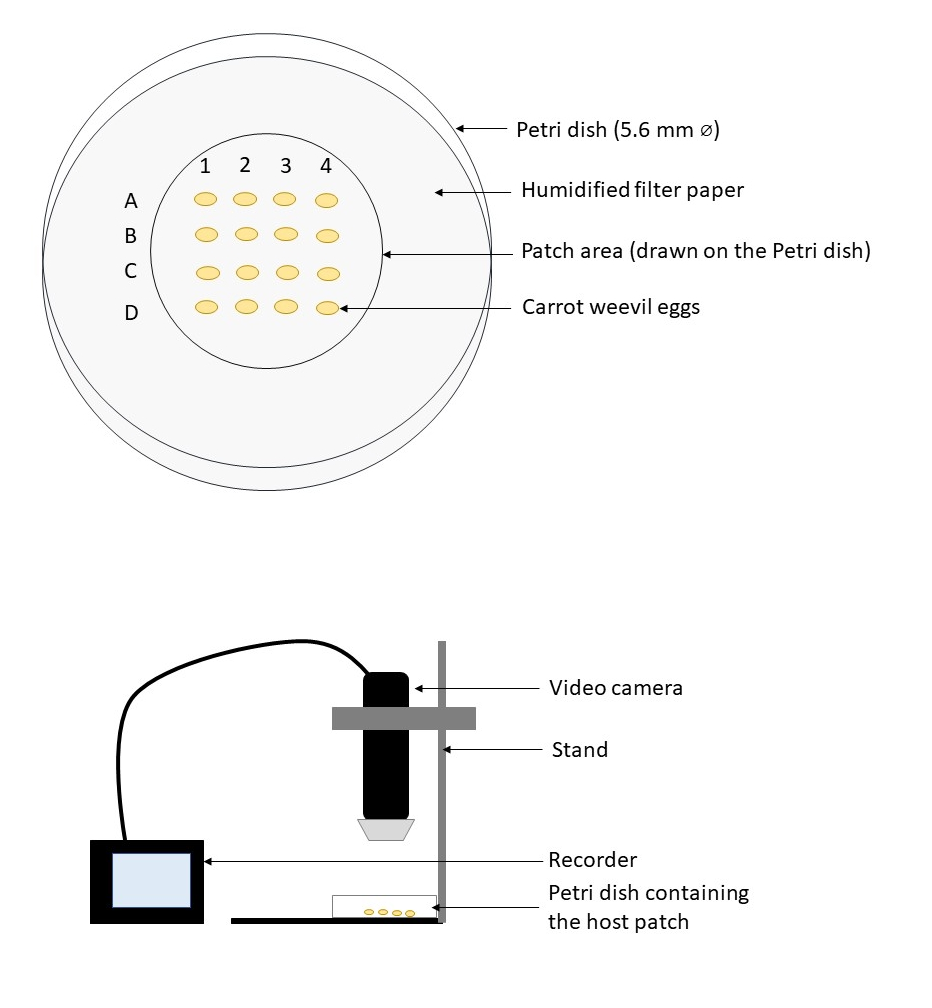

Supplement: S2 Fig — (TIF) [file pone.0254750.s002.tif]
